# The impact of threshing unit structure and parameters on enhancing rice threshing performance

**DOI:** 10.1038/s41598-025-91118-5

**Published:** 2025-02-20

**Authors:** Mohamed Anwer Abdeen, Weibin Wu, Abouelnadar El. Salem, Ahmed Elbeltagi, Ali Salem, Khaled A. Metwally, Guozhong Zhang, Abdallah Elshawadfy Elwakeel

**Affiliations:** 1 College of Engineering, South China Agricultural University, Guangzhou, 510642 China; 2 Agricultural Engineering Department, College of Agriculture, Zagazig University, Zagazig, 44519 Egypt; 3 Yellow River Delta Intelligent Agricultural Equipment Industry Academy, Dong Ying, China; 4 Desert Research Center, Mataria, 11753 Egypt; 5 Agricultural Engineering Department, Faculty of Agriculture, Mansoura University, Mansoura, 35516 Egypt; 6 Civil Engineering Department, Faculty of Engineering, Minia University, Minia, 61111 Egypt; 7 Structural Diagnostics and Analysis Research Group, Faculty of Engineering and Information Technology, University of Pécs, Pécs, 7622 Hungary; 8 Soil and Water Sciences Department, Faculty of Technology and Development, Zagazig University, 44511 Zagazig, Egypt; 9 College of Engineering, Huazhong Agricultural University, Wuhan, 430070 China; 10 Agricultural Engineering Department, Faculty of Agriculture and Natural Resources, Aswan University, Aswan, 81528 Egypt

**Keywords:** Threshing efficiency, Rice combines, Axial flow thresher, Rice threshing, Threshing power., Plant sciences, Energy harvesting, Mechanical engineering

## Abstract

Improving rice threshing unit performance and structure significantly enhances the thresher’s overall efficiency by raising its productivity while lowering costs and power consumption. This study evaluated and optimized longitudinal axial flow threshing device performance using two threshers’ structures, namely conical thresher and drum thresher, under different rotating speeds of 1100, 1300, and 1500 rpm and feeding rates of 0.8, 1.1, and 1.4 kg/s. The studied parameters were evaluated regarding thresher throughput, efficiency, seed damage, and specific energy, and it was figured that the experimental parameters greatly affected the whole performance of the threshing unit. The drum thresher increased the threshing throughput from 2304 to 2448 kg/h and maximized the efficiency from 98.6 to 99.07% at 1500 rpm rotation speed and 1.8 kg/s feeding rate. Moreover, it reduced the specific energy from 3.37 to 3.15 kW.h/ton for the experimental variables 1100 rpm speed and 1.4 kg/s feeding. The lowest damage rate of 0.24% was recorded using the drum thresher at a feeding rate of 0.8 kg/s and 1100 rotating speed. These results highlight the distinct effect of the new thresher structure and operating parameters on the rice threshing device’s overall effectiveness for medium-sized combine harvesters.

## Introduction

Rice and wheat are essential staples for 95% of the global population. With half the world’s people relying on rice, it is the second most important grain crop, following wheat. The rice yield significantly impacts regional development, making it a crucial element in food security and economic stability^1–3^.

Grain harvesters are used to harvest many crops under different operating and environmental conditions as they help reduce labor and maximize the threshing efficiency^4,5^. Recently, mature rice crops have been mainly harvested by combine harvester^6^, which efficiently combines the processes of harvesting, threshing, and cleaning the grain in one operation^7^.

Postharvest practices were found to have a great influence on enhancing the quality of agricultural products^8^. Grains are being threshed inside the threshing gap by extrusion and bending forces^9,10^. The kernels’ separating rate is the speed at which intact kernels are extruded during the threshing process^11^. The threshing device is the primary power consumer among combine harvester units, so lowering its power consumption can help develop a lightweight, high threshing efficiency and high feeding combine harvester^12^. The threshing device plays a crucial role in the combine’s performance, impacting threshing efficiency while ensuring maximum yield with optimal separation and minimal seed loss^13,14^.

Threshers are typically classified based on the movement of the crop inside the threshing chamber into two main types: axial and cross-flow threshers. In a cross-flow thresher, the crop is threshed as it moves transversely through the threshing gap. In contrast, an axial flow thresher processes the crop along its axis, using the impact force generated by the threshing cylinder to complete the threshing process^15^.

In axial flow threshers, the harvested crop moves helically along the cylinder’s axis, experiencing a longer threshing period due to the repeated impact of the bars^16^. In axial flow threshers, the seeds are separated by rubbing against each rather than being struck by the threshing elements as in a cross flow threshers, which helps protect them from damage^17^. Chethan et al.^18^ investigated the crop flow path in axial flow paddy threshers, highlighting the superior performance of axial flow over cross-flow threshers in terms of handling larger volumes of crops and providing better separation of grains from the straw.

The longitudinal axial flow separation device is generally used in combine harvesters as it has more threshing time, a smoother process, and better adaptability^19–22^. However, it has certain drawbacks, including high power consumption, broken stems, and high impurity content, which require more research and study. Threshers are also categorized by the type of the threshing cylinder: spike-toothed and rasp bars. The spike-toothed thresher uses a striking action to thresh the crop, whereas the rasp bar employs rubbing and friction. The spike-toothed threshers provide a superior threshing effect compared to rasp bars, achieving higher productivity across various rotating speed^23^.

The threshing performance is affected by many factors: crop moisture content, threshing cylinder diameter, thresher type, threshing spike shape, spike number and size, concave clearance, feeding rate, and threshing cylinder speed^24^. Many scholars suggested enhancing the threshing effect by optimizing the threshing gap, speed, and threshing cylinder diameter^25,26^.

The feeding rate and thresher length greatly impact determining the grain density and volume within the threshing gap. Therefore, selecting an appropriate feeding method is essential to ensure efficient and effective threshing. Optimizing these parameters can enhance the overall performance of the thresher and maintain high-quality grain output^27^. Increasing the thresher feed rate and rotation speed led to higher power requirements due to the increased load from excessive stalks in the threshing gap. However, this also enhanced productivity, as a greater rice mass was processed in a certain time^28^.

Increasing the thresher rotating speed increased the total efficiency of the threshing unit^29^. Singh et al.,^30^ stated that threshing efficiency positively correlated with the thresher rotating speed. Feeding rate, concave clearance, cylinder speed, and crop variety all favorably impact threshing efficiency^31^. The higher drum speed decreased threshing losses but increased damaged grain because of the spikes’ increased impact on the crop stalks^32^. Increasing the thresher’s speed resulted in more impact on the spikes and raised the threshing efficiency. The maximum threshing efficiency of 99.76% was achieved under 0.25 kg/s feeding rate and 1400 rpm drum speed^33^.

The study seeks to enhance rice crop production, minimize labor and power required, and enhance the quality of rice grains. To achieve these aims, it is crucial to test, develop, and optimize a rice threshing unit. Consequently, this paper optimized a longitudinal axial flow threshing unit for rice combine and creatively constructed and designed an axial flow drum-shaped thresher with higher efficiency, higher productivity, and lower energy requirements and damage. This drum thresher can process a larger volume of crops in a shorter time. Besides, its smooth threshing action can reduce grain damage, resulting in higher productivity and quality.

## Materials and methods

### Experimental platform

**Fig. 1:**
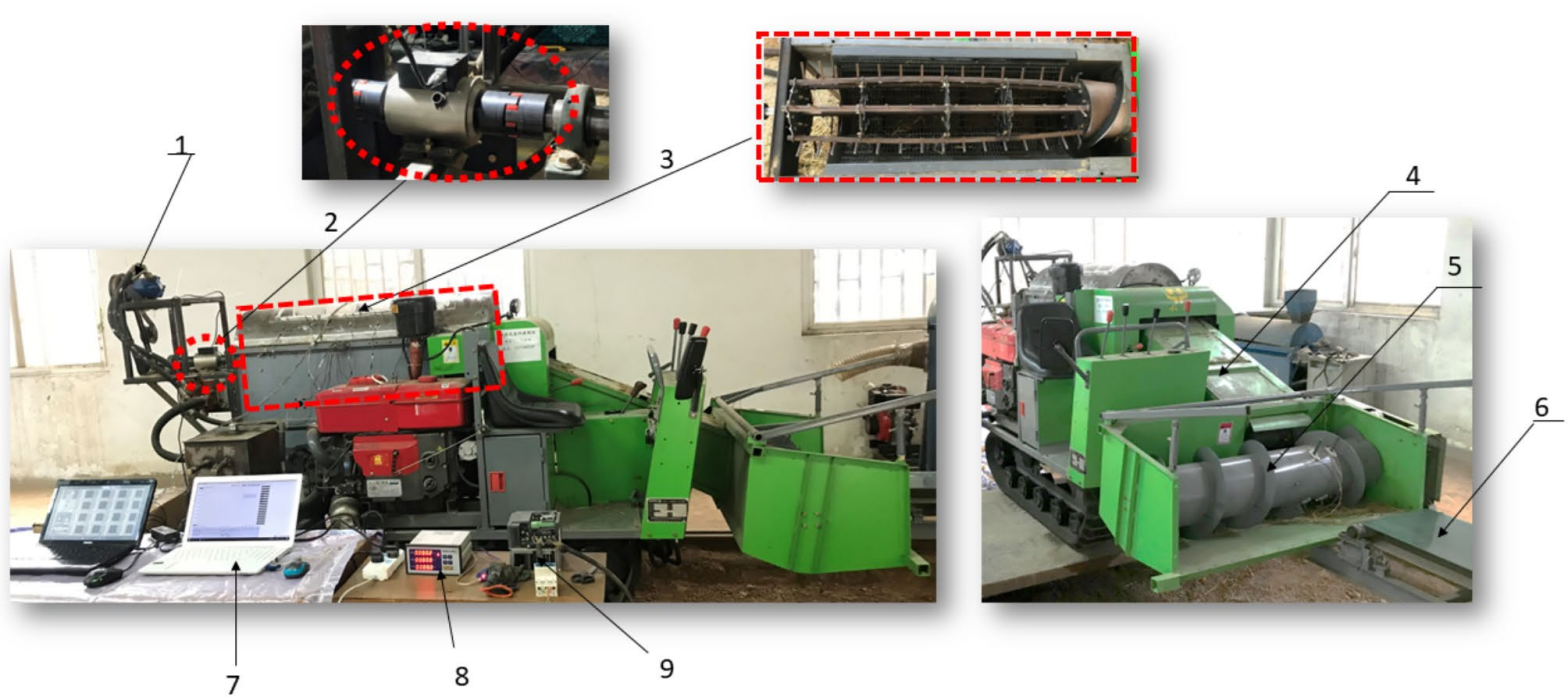
Testing platform. (1) Hydraulic, (2) Torque sensor, (3) Threshing and separation device, (4) Conveyor chain, (5) Feeding auger, (6) Feeding device, (7) Laptop, (8) Torque sensor acquisition unit, (9) Frequency converter.

A longitudinal axial flow platform (Fig. 1) with dimensions of 3700 mm, 1460 mm, and 1540 mm for length, width, and height has been constructed to optimize the threshing device’s performance and structure. The platform comprises a feeder composed of a revolving rubber belt with dimensions of 6000 mm in length and 50 mm in width, driven by an electric motor and controlled for speed by a frequency converter. This feeder transports rice to the threshing unit through a rotating auger.

A torque sensor was installed on the rear end of the threshing shaft to compute the threshing power, torque, and rotating speed. A diesel engine powered the machine, and the power was conveyed to the rotating parts using a belt and pulley.

### Threshing unit

The threshing unit contains a thresher, a cover, and a concave. The thresher consists of a feeding screw auger, six threshing bars, spike teeth, and discs which connect the thresher parts. Two kinds of thresher structures were introduced in the study (conical thresher and drum thresher).

**Fig. 2:**
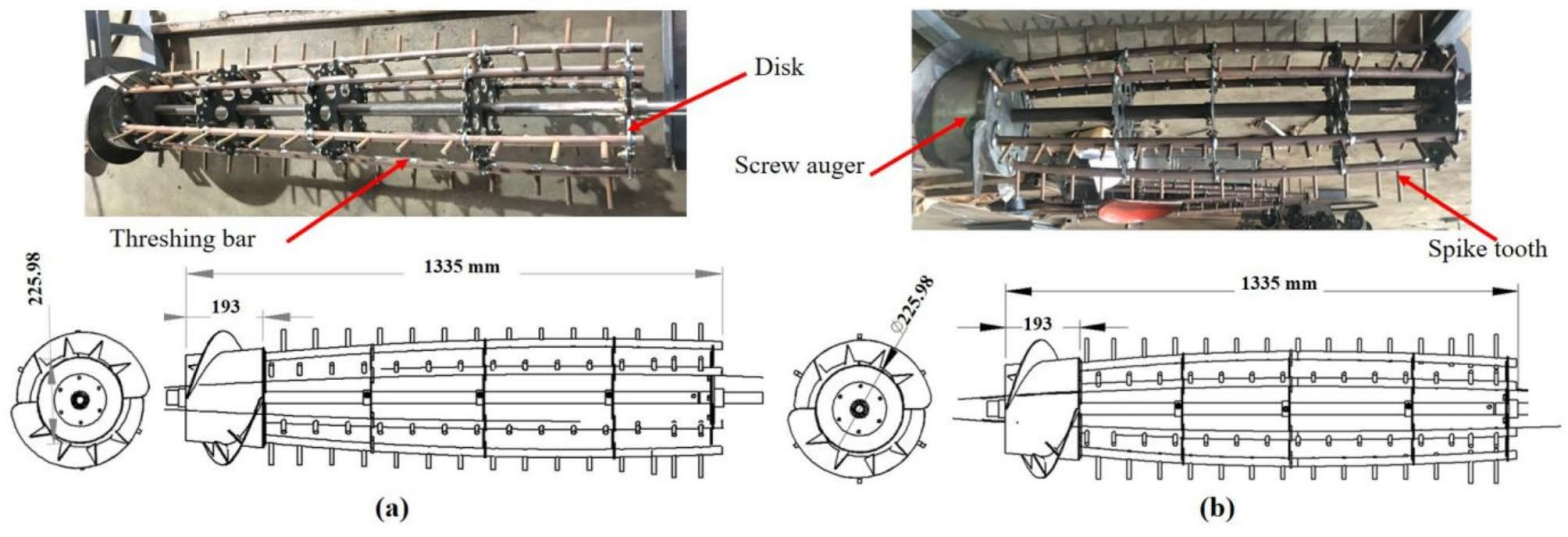
Longitudinal axial flow threshers; (**a**) conical thresher; (**b**) drum thresher.

The newly designed drum thresher dimensions are 370 mm in diameter and 1360 mm in length. The threshing tooth height ranges from 50 to  70 mm, the total number of rod teeth was 87, and the distance between two adjacent teeth was 40 mm. Thresher structures are shown in Fig. 2. The thresher structure can be easily modified, as the threshing bars are connected to the auger and disks using bolts and nuts. This flexibility makes the thresher suitable for a variety of crops and helps save costs.

The crop properties have been measured and are shown in Table 1, according to the study of^34^.

**Table 1:** Rice properties.

Property	Value
Average Grain Length (mm)	9.75
Average Grain Width (mm)	2.75
Average Grain Thickness (mm)	2.02
Grain Moisture Content (%)	13.32
1000 Grains Weight (kg)	0.0304
Average Stalk Length (mm)	964.08
Stalk Moisture Content (%)	63.21
Average spike length (mm)	20.85
Max Shearing Force (N)	249.6
Max Bending Force (N)	9

### Experimental design

Experimental design methods might be used to analyze the threshing machine’s performance as it reduces the effects of uncontrolled factors and minimizes the test number^35,36^.

The full factorial design was utilized for the experiment using Minitab 18 software (Table 2), and the impact of the studied parameters on the threshing machine performance was analyzed using the same technique: the two-way analysis of variance (ANOVA).

**Table 2:** Experiment full factorial design.

Factor	Levels	Values
Thresher type	2	Conical, Drum
Thresher speed, rpm	3	1100, 1300, 1500
Feeding rate, kg/s	3	0.8, 1.1, 1.4

The threshing machine’s performance was assessed by investigating its response to different threshing cylinder types, namely the cylindrical and drum thresher. The performance was also evaluated under various feeding rates of 0.8, 1.1, and 1.4 kg/s and thresher speeds of 1100, 1300, and 1500 rpm. These parameter values were chosen based on the methodology outlined in^34,37^. Before the experiments, the rice crop was harvested from a field in Wuhan and then transported to the Huazhong Agricultural University workshop, where the platform was installed. The rice crop was uniformly distributed on the feeding belt for the last five meters, leaving the first meter empty to guarantee uniform feeding. The feeding rate was controlled by changing the feeding device speed using the frequency converter, while the thresher revolving speed was changed utilizing different belts and pulleys.

## Measurements

### Thresher throughput

Thresher throughput is the material quantity passes per time unit. It depends on the threshing rate, grains passing through the concave holes in the time unit, and the crop retaining time inside the threshing gap.

The throughput of the thresher was calculated by dividing the total threshed rice seeds by their threshing time, as follows:

1 $$\:Throughput\:=\:\frac{Total\:weight\:of\:threshed\:seed\left(g\right)\:}{Total\:threshing\:time\left(s\right)}$$

### Threshing efficiency

The threshing efficiency percentage was calculated according to the following Eq. (3)^2^:

2 $$\:Threshing\:efficiency\:=\:\frac{Weight\:of\:threshed\:seeds\:\left(g\right)\:}{Total\:weight\:of\:seeds\:in\:sample\:\left(g\right)}\:\times\:\:100$$

### Seed damage

The percentage of damaged seeds was obtained according to the following equation:

3 $$\:Seed\:damage\:\left(\%\right)\:=\frac{Ds}{Ts}\times\:100$$

Where Ds is the weight of damaged seeds in g, and Ts is the total sample seeds mass (g).

### Specific energy

The consumed threshing power was derived from the data collected by the torque sensor installed on the threshing drum shaft. Then, the power was divided by machine output to calculate the specific energy (kW.h/ton).

4 $$\:Specific\:energy\:(kW.h/ton)\:=\:\frac{Power\:\left(kW\right)}{Throughput\:\left(\frac{Ton}{h}\right)}$$

## Results and discussions

### Analysis of variance

In this study, a 5% significance level ANOVA was performed using Minitab 18 software full factorial design analysis to analyze the study parameters’ effect on the threshing performance. The results are illustrated in (Tables 3, 4, 5 and 6), which also show the contribution rate of each factor on threshing performance.

P-values indicate the significant impact of each parameter on the results. Meanwhile, the contribution rate ranks the parameters by their influence, with the highest contribution rate indicating the most impactful parameter on the results. Following the ANOVA analysis, High-accuracy 3D graphs were drawn using the OriginPro 2022 and Minitab 18 software to illustrate the effect of the studied parameters on the thresher performance.

Table 3 shows that thresher speed is the most critical factor affecting threshing machine throughput, with a contribution rate of 63.82%, followed by feeding rate and thresher type. P-Values demonstrate the significance of all the parameters studied in the results.

**Table 3:** Throughput ANOVA.

Source	DF	Seq SS	Contribution (%)	Adj SS	Adj MS	F-Value	*P*-Value
Thresher type	1	165,888	6.96	165,888	165,888	16.82	0.001
Thresher speed	2	1,521,936	63.82	1,521,936	760,968	77.15	0.000
Feeding rate	2	578,448	24.26	578,448	289,224	29.32	0.000
Error	12	118,368	4.96	118,368	9864		
Total	17	2,384,640	100.00				

Table 4 Revealed a significant effect on the experimental results for all parameters as the P-values are lower than 0.05. The table also shows that the most influential factor in threshing efficiency is feeding rate (60.06% contribution), followed by thresher type, and finally, the thresher speed. The higher contribution rate reflects that this parameter is the most influential on the results.

**Table 4:** Efficiency ANOVA.

Source	DF	Seq SS	Contribution (%)	Adj SS	Adj MS	F-Value	*P*-Value
Thresher type	1	0.68135	19.66	0.68135	0.68135	90.94	0.000
Thresher speed	2	0.61331	17.69	0.61331	0.30666	40.93	0.000
Feeding rate	2	2.08183	60.06	2.08183	1.04092	138.93	0.000
Error	12	0.08991	2.59	0.08991	0.00749		
Total	17	3.46640	100.00				

The thresher type had the greatest effect on damage rate, with a rate of 56.15%, followed by the feeding rate and thresher speed (Table 5). the ANOVA analysis P-values illustrates the significant effect of each experimental variable on the threshing process.

**Table 5:** Damage rate ANOVA.

Source	DF	Seq SS	Contribution (%)	Adj SS	Adj MS	F-Value	*P*-Value
Thresher type	1	0.44809	56.15	0.44809	0.448089	313.84	0.000
Thresher speed	2	0.15968	20.01	0.15968	0.079839	55.92	0.000
Feeding rate	2	0.17308	21.69	0.17308	0.086539	60.61	0.000
Error	12	0.01713	2.15	0.01713	0.001428		
Total	17	0.79798	100.00				

Table 6 Illustrates that the thresher type influences specific energy most, followed by feeding rate and thresher speed. Meanwhile, all the parameters had a significant influence on the results.

**Table 6:** Specific Energy ANOVA.

Source	DF	Seq SS	Contribution (%)	Adj SS	Adj MS	F-Value	*P*-Value
Thresher type	1	5.718	31.78	5.718	5.7183	29.87	0.000
Thresher speed	2	4.349	24.17	4.349	2.1743	11.36	0.002
Feeding rate	2	5.629	31.29	5.629	2.8147	14.70	0.001
Error	12	2.297	12.77	2.297	0.1914		
Total	17	17.993	100.00				

### Effect of experimental variables on thresher throughput

**Fig. 3:**
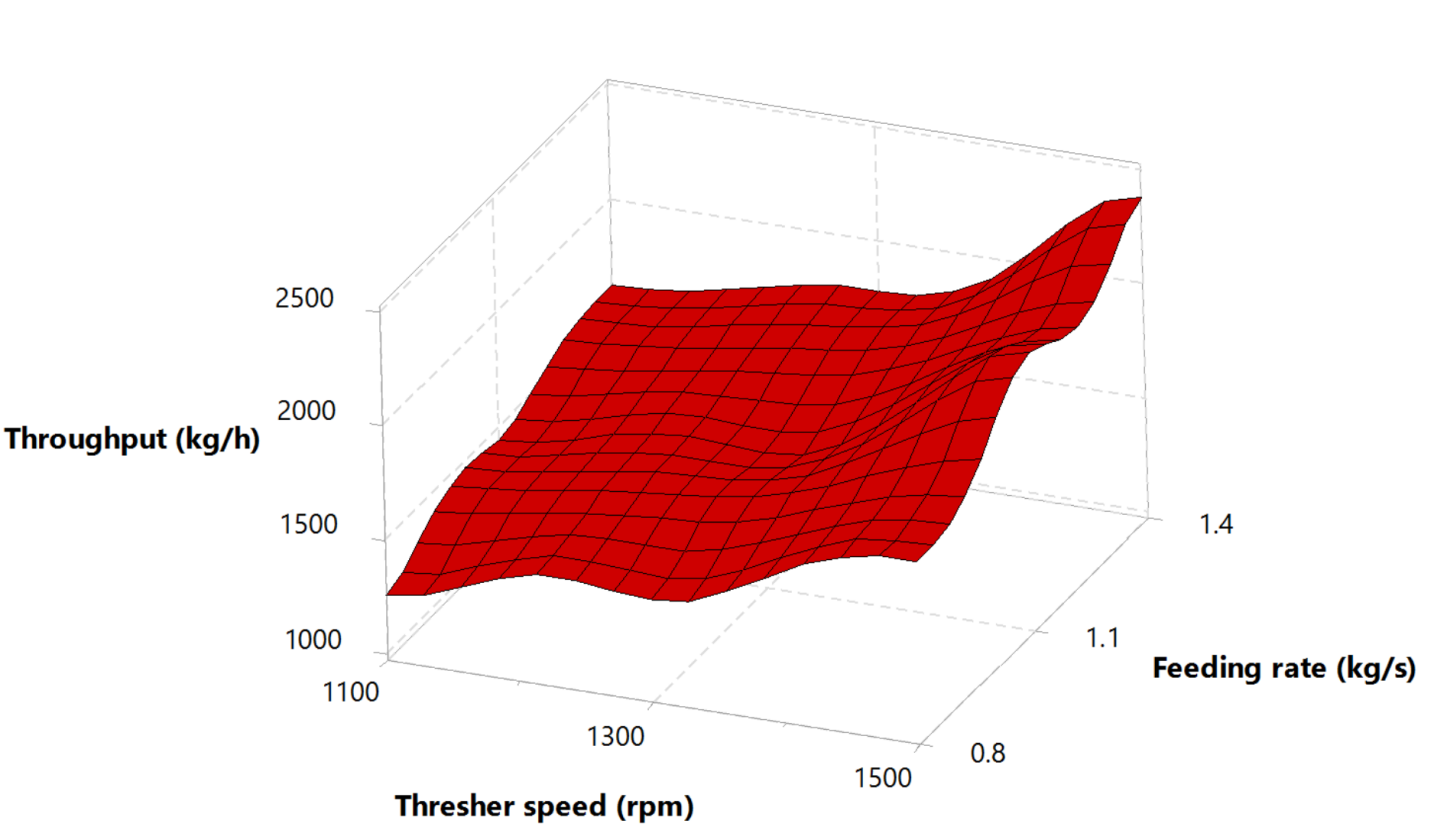
Thresher throughput versus thresher speed and feeding rate.

Throughput would increase with an increase in the operating speed of a thresher, as reported by^38^. The thresher throughput (Fig. 3) illustrated an increase while the thresher speed and feeding rate increased for both thresher structures, agreeing with the results of^28,39^. The increase in throughput can likely be attributed to the larger mass of the threshed mixture when operating at a higher feed rate, combined with the reduced threshing time that comes from increasing the rotating speed.

**Fig. 4:**
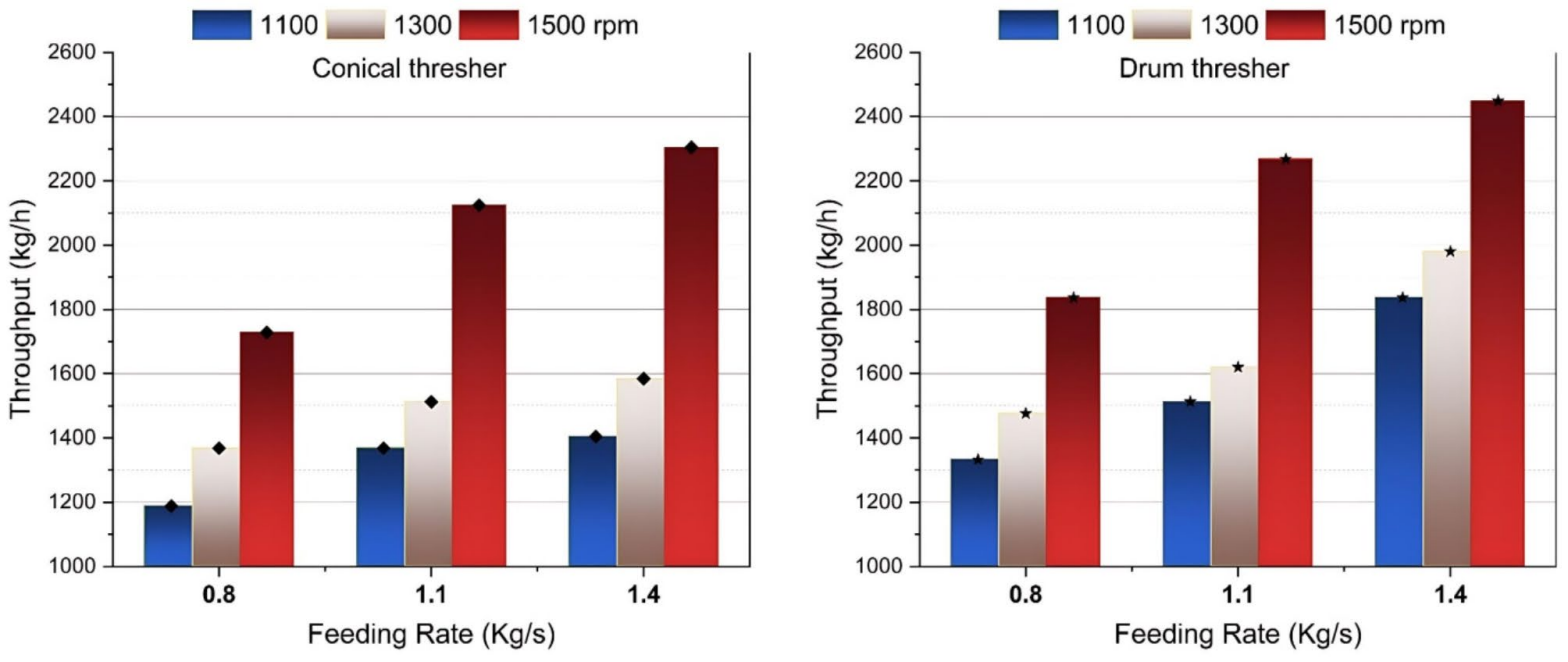
Effect of study parameters on thresher throughput.

The drum thresher demonstrated higher productivity than the conical thresher, as shown in Fig. 4. This increased productivity is likely attributed to the increasing clearance at the drum thresher’s end, resulting from its smaller diameter, which smoothens and accelerates the discharging process of the threshed mixture from the threshing gap to the outlet while reducing the friction between the thresher parts and the moving mixture, thus reducing the overall threshing time. The highest throughput achieved was 2448 kg/h, recorded with the drum thresher operating at a thresher speed of 1500 rpm and a feeding rate of 1.4 kg/s, representing an increase of more than 6.25% and highlighting the efficiency and capability of the drum thresher in processing large crop quantities effectively.

### Effect of experimental variables on the threshing efficiency

**Fig. 5:**
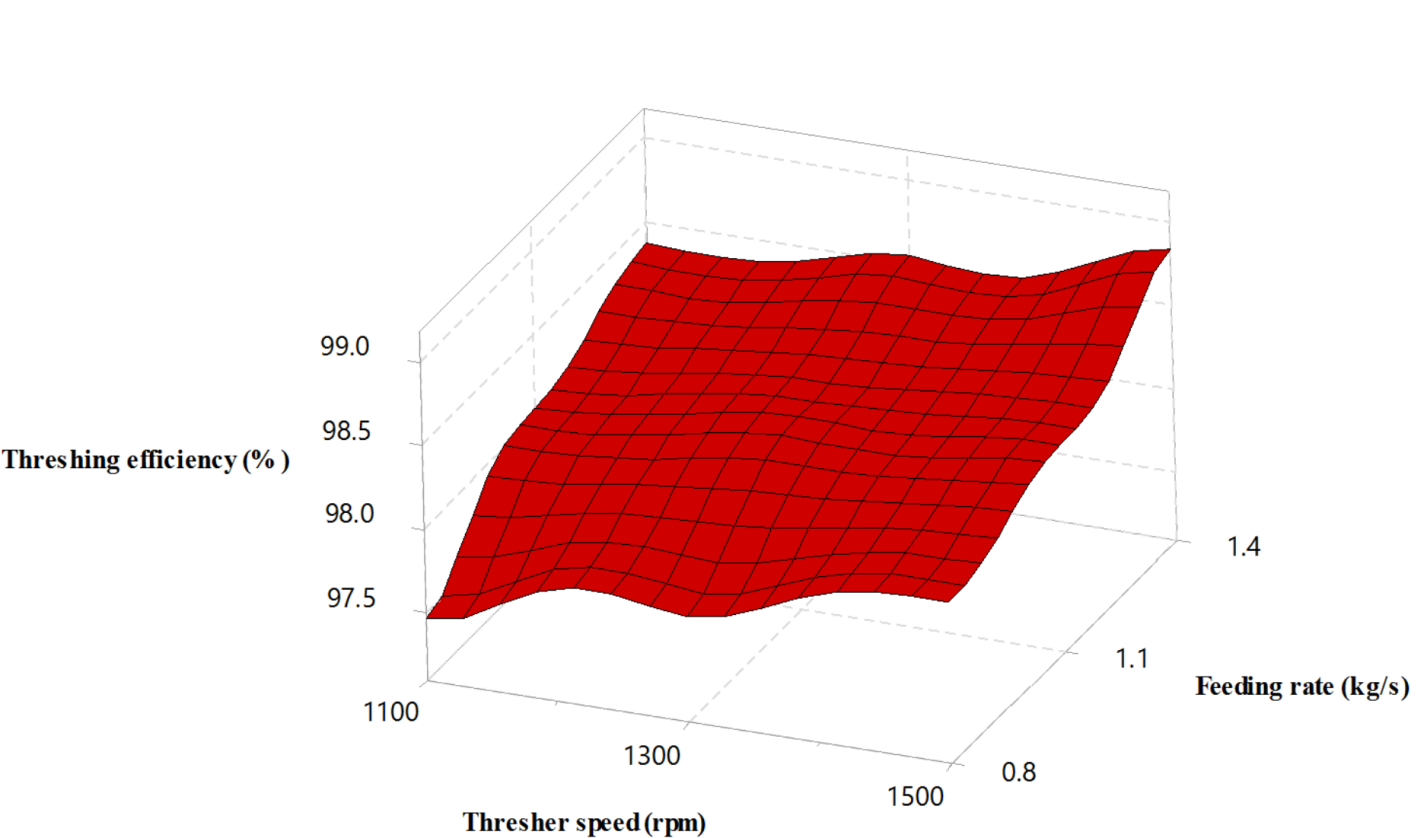
Threshing efficiency versus thresher speed and feeding rate.

Figure 5 shows that increasing the thresher’s feeding rate and rotating speed increased the threshing efficiency for both threshers, which agreed with the results of^39,40^. These results could be attributed to the high collision frequency of spikes hitting rice ears and the increased friction between grain and threshing concave resulting from the increasing rotation speed. Additionally, the increased feed rate caused excessive crop mass to pass through the threshing gap per unit of time.

**Fig. 6:**
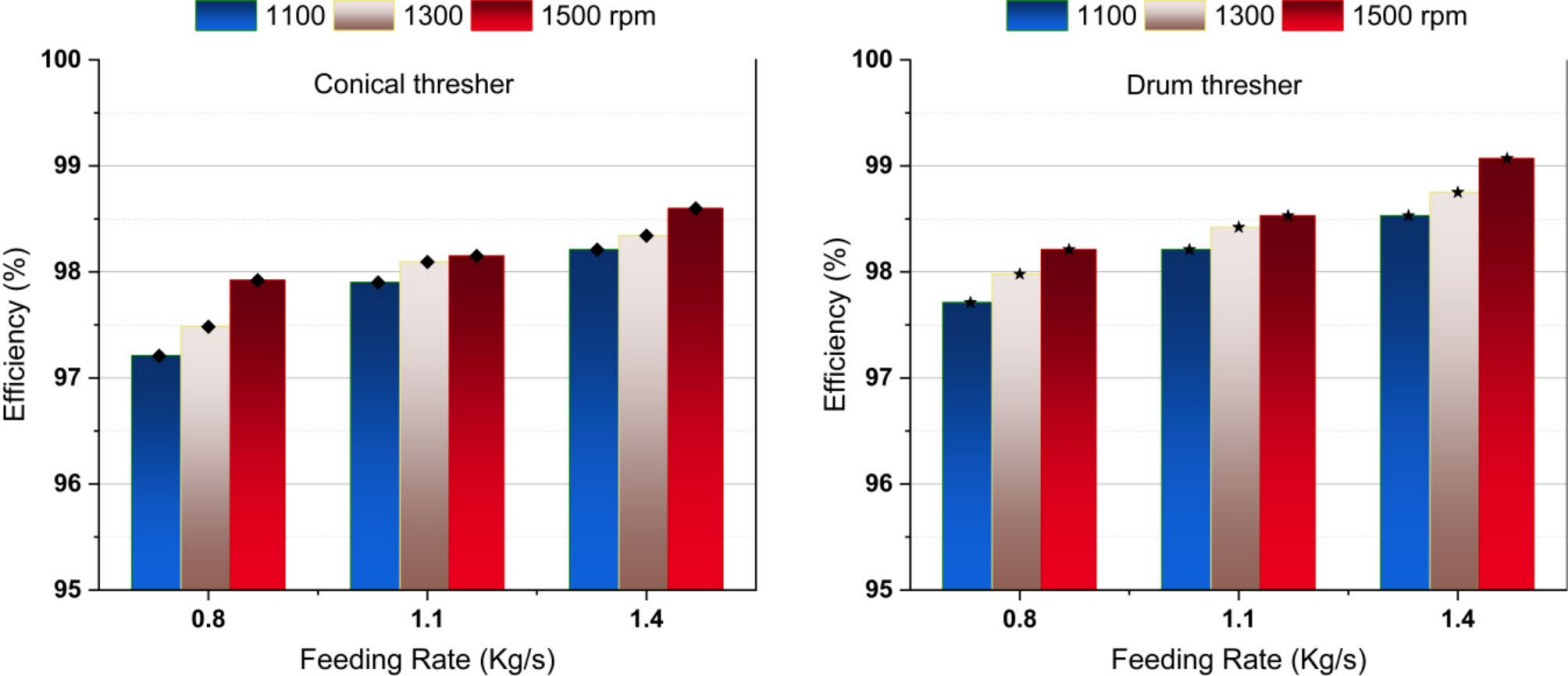
Effect of study parameters on threshing efficiency.

Notably, the drum thresher recorded higher threshing efficiency for all the experimental variables, as seen in Fig. 6. This could be attributed to the curved design of the drum thresher bars, which smooths the passage of the mixture in the threshing gap and reduces the separation time. The maximum threshing efficiency (99.07%) was obtained using a drum thresher at a feeding rate of 1.4 kg/s and 1500 rpm rotating speed.

### Effect of experimental variables on the damage rate

**Fig. 7:**
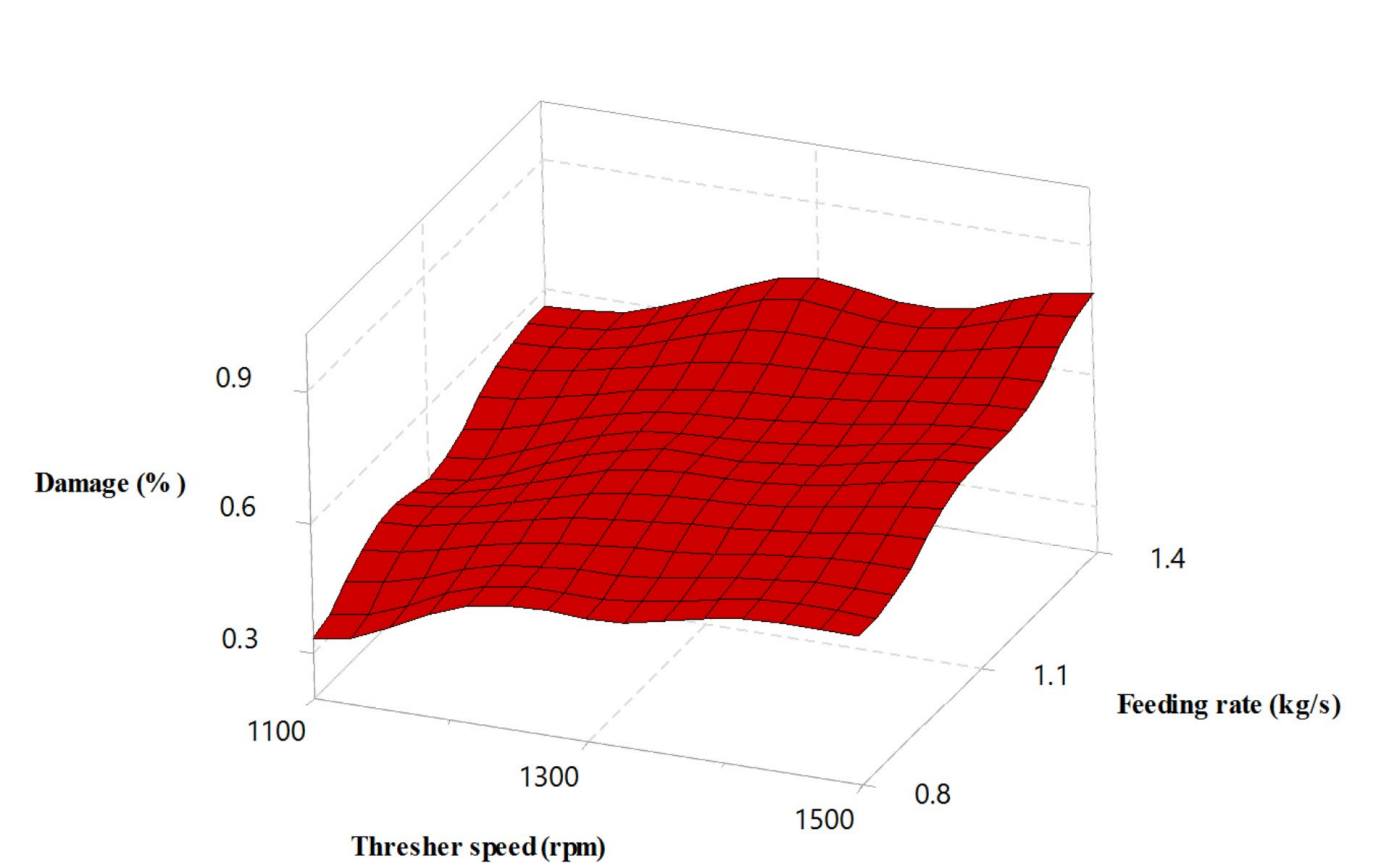
Seed damage versus thresher speed and feeding rate.

**Fig. 8:**
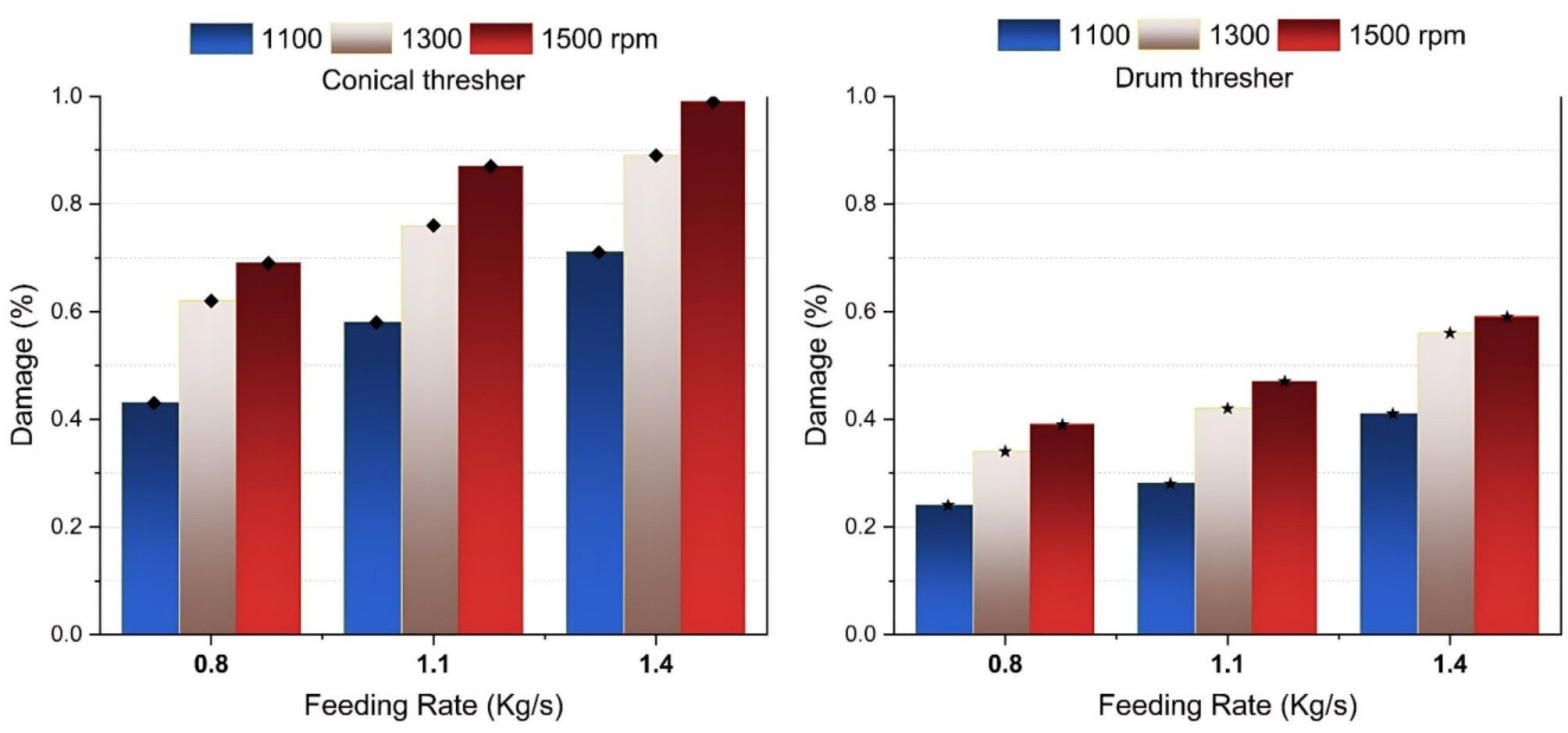
Effect of study parameters on seed damage.

The increase in rotor speed and feeding rate led to a higher seed damage ratio as the crop density in the threshing gap increased, and the impact from the thresher tooth on the crop mixture intensified (Figs. 7 and 8). These results agreed with^41–45^.

The lowest achieved damage rate was 0.24%, significantly lower than rates reported by some other researchers. For example, Esgici et al.^44^ reported a broken grain rate of 6.876%, Hailemesikel et al.^45^ recorded 1.3%, and Bhardwaj et al.^17^ recorded 4.00%.

The drum-shaped thresher resulted in 16.56% fewer damaged seeds than the conical thresher. This result can be attributed to the grains’ reduced rubbing and grinding action at the beginning and the end of the threshing gap, thanks to the increased threshing clearance. Additionally, the rice panicles had more buffer space, contributing to a lower damage rate.

### Effect of experimental variables on specific energy

**Fig. 9:**
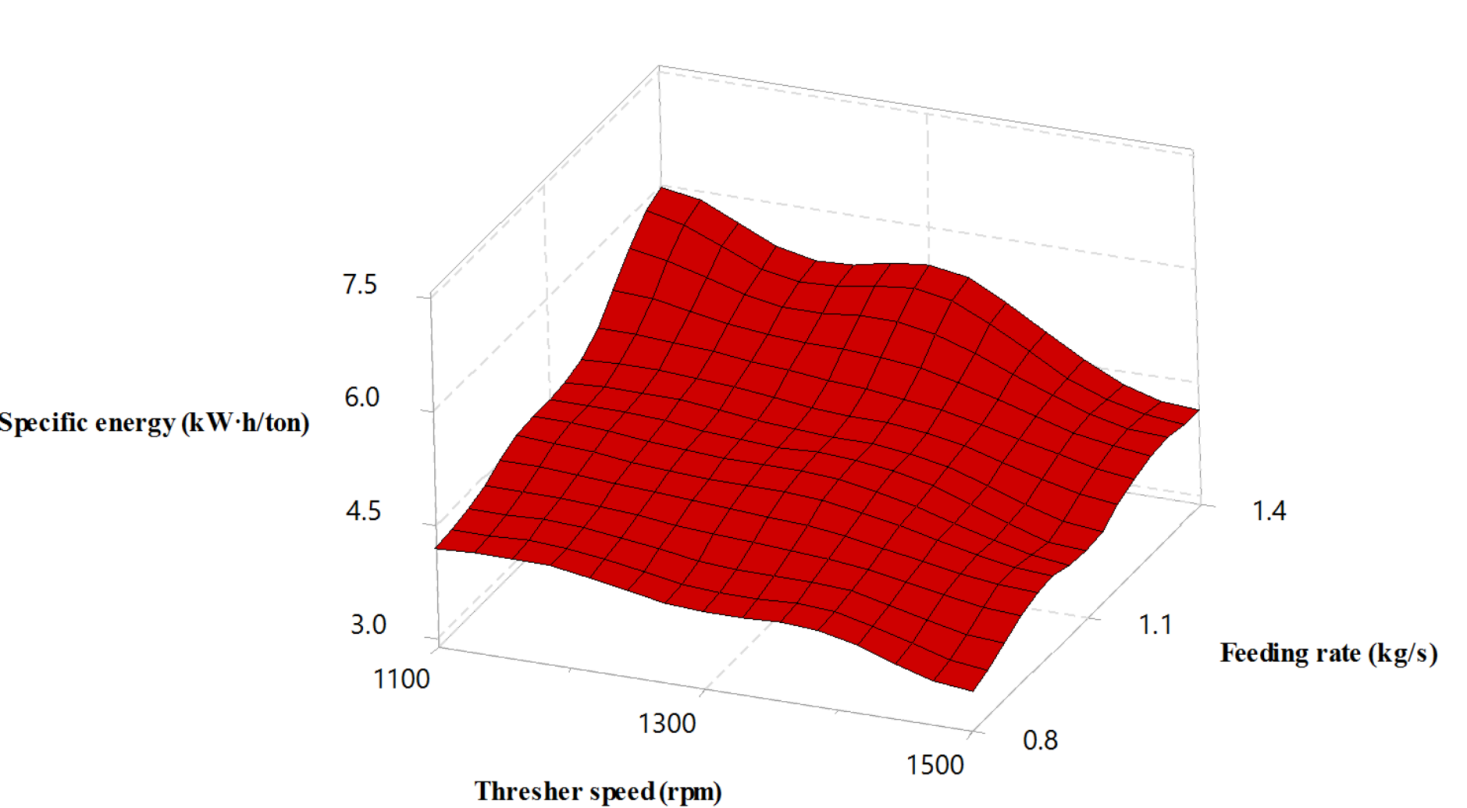
Thresher specific energy versus thresher speed and feeding rate.

The specific energy of the thresher was calculated based on its productivity and required power during the threshing process. Figure 9 illustrates a decrease in the specific energy while increasing the rotor speed, which can be attributed to the dominant enhanced productivity of the thresher compared to the increased power in agreement with^46,47^. Besides, the specific energy increased while the thresher feeding rate increased. These findings reflect the excessive crop high load on the thresher while increasing the feeding amount. These results agreed with those obtained by Asli-Ardeh et al.^28^, who noticed a significant effect of the drum speed on the power and energy requirements.

**Fig. 10:**
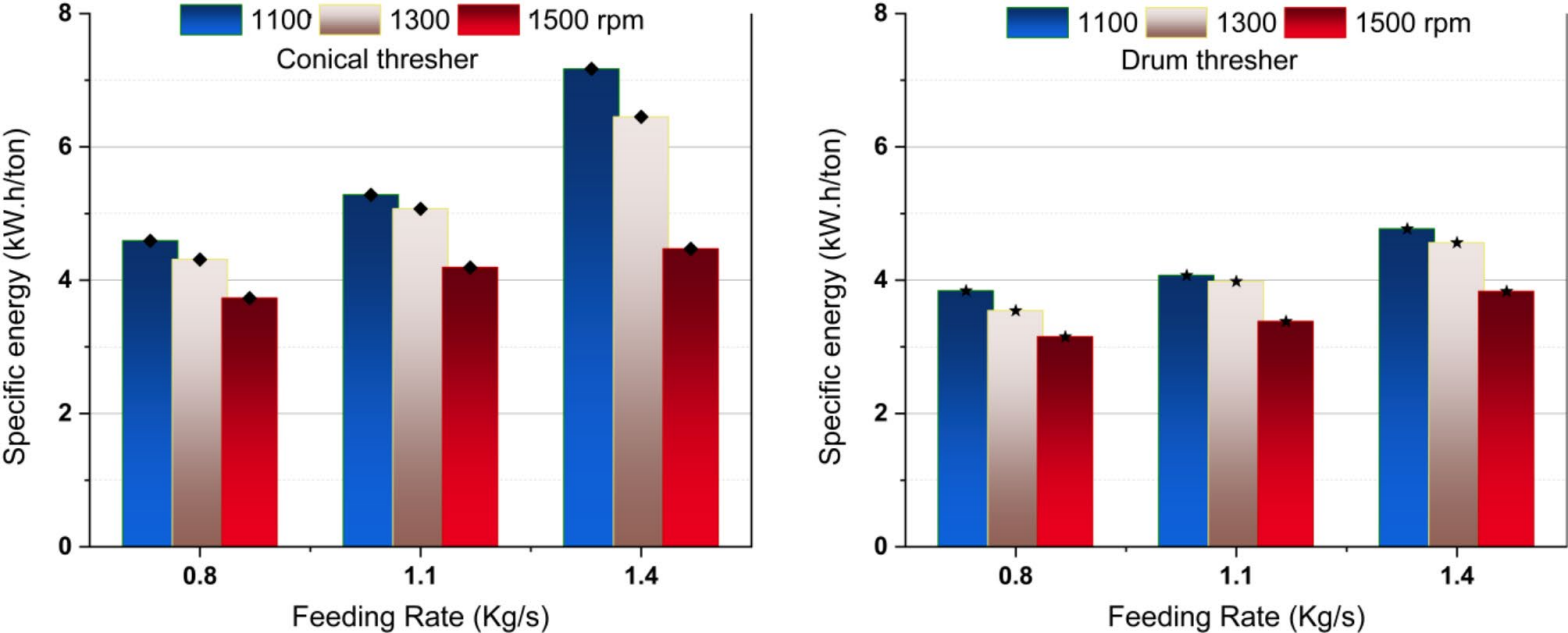
Effect of study parameters on specific energy.

Using the drum-shaped thresher resulted in over a 15% reduction in specific energy consumption. This may be due to the drum thresher’s lower crop resistance resulting from the adjustable threshing gap and the smooth flow of the threshed mixture, which reduces the load on the drive engine and lowers power consumption. The lowest specific energy recorded was 3.15, achieved with the drum thresher operating at a rotation speed of 1500 rpm and a feeding rate of 0.8 kg/s (see Fig. 10). This significant reduction highlights the efficiency and energy-saving benefits of the drum-shaped thresher.

In order to statistically analyze the results and identify whether significant differences exist between the experimental variables, Minitab 18 was used to conduct a Tukey pairwise comparison (Table 7).

The mean and grouping values revealed significant differences, illustrating that the drum thresher outperformed the conical thresher across all measured results.

**Table 7:** Tukey pairwise analysis for experimental variables.

Parameter	Value	Throughput		Efficiency		Damage		Specific energy	
		Mean	Grouping	Mean	Grouping	Mean	Grouping	Mean	Grouping
Thresher type	Drum	1812	A	98.37	A	4.51	B	3.89	B
	Conical	1620	B	97.98	B	2.77	A	5.02	A
Thresher speed	1100	1440	B	97.96	C	0.44	C	4.95	A
	1300	1590	B	98.17	B	0.59	B	4.64	A
	1500	2118	A	98.41	A	0.66	A	3.79	B
Feed rate	0.8	1488	C	97.75	C	0.45	C	3.65	C
	1.1	1734	B	98.21	B	0.56	B	4.32	B
	1.4	1926	A	98.58	A	0.69	A	5.20	A

*While grouping analysis, each parameter is treated as an independent unit. Different letters indicate significant differences; a higher mean value reflects higher significance and impact.

## Conclusions

This study focused on optimizing the performance of two longitudinal flow threshing devices for rice combines: conical-shaped and drum-shaped threshers. The threshing devices were tested at various speeds of (1100, 1300, and 1500 rpm) and feeding rates of (0.8, 1.1, and 1.4 kg/s). The study examined the impact of the experimental variables on thresher productivity, threshing efficiency, specific energy consumption, and damage rate. The findings revealed a positive correlation between the feeding rate and rotation speed with all the measured parameters.

The drum thresher outperformed the conical thresher, leading to higher productivity by 6.25%, improved efficiency by 0.5%, reduced damage by 44.18%, and reduced energy consumption by 6.52% across all the experimental variables. The drum thresher achieved the highest threshing efficiency and throughput of 99.07% and 2448 kg/h, respectively, when operated at a rotating speed of 1500 rpm and a feeding rate of 1.4 kg/s. Conversely, the lowest specific energy of 3.15 kW.h/kg and the lowest damage ratio of 0.24% were recorded when the drum thresher was operated at 1100 rpm rotating speed and a feeding rate of 0.8 kg/s.

This study introduced an innovative thresher design that noticeably enhanced the threshing machine’s performance as it achieved lower threshing energy consumption, lower damage rate, increased productivity, and improved efficiency. It made the threshing process more sustainable and cost-effective and enhanced agricultural productivity.

The authors aim to conduct further experiments to investigate the threshing of various rice varieties under different moisture contents. Additionally, we plan to test and optimize the designed threshers to efficiently thresh other crops, such as wheat and sunflower, by adjusting the concave clearance and openings, replacing the tooth bars with rasp bars (as they can be changed easily), and adjusting the rotor speed and feeding rate.

We may also mention that additional efforts are needed for field operations to overcome the variable feeding rate, as the feeding height of the crop will change due to uneven field levels and machine vibrations. By expanding the scope of our research, we hope to enhance the versatility and efficiency of our threshers, ultimately benefiting a wider range of agricultural applications.

## Data Availability

The datasets used and/or analysed during the current study available from the corresponding author on reasonable request.

## References

[1] Cordero-Lara, K. I. Temperate Japonica rice (Oryza sativa L.) breeding: History, present and future challenges. *Chil. J. Agricultural Res.* **80**, 303–314. 10.4067/S0718-58392020000200303 (2020).

[2] Dale, D. S., Liang, L., Zhong, L., Reba, M. L. & Runkle, B. R. K. Deep learning solutions for mapping contour levee rice production systems from very high resolution imagery. *Comput. Electron. Agric.* **211**, 107954. 10.1016/j.compag.2023.107954 (2023).

[3] Dhillon, A. K., Sharma, N., Dosanjh, N. K., Goyal, M. & Mahajan, G. Variation in the nutritional quality of rice straw and grain in response to different nitrogen levels. *J. Plant Nutr.* **41**, 1946–1956. 10.1080/01904167.2018.1482915 (2018).

[4] Unakıtan, G. & Aydın, B. A comparison of energy use efficiency and economic analysis of wheat and sunflower production in Turkey: A case study in Thrace region. *J. Energy* **149**, 279–285. 10.1016/j.energy.2018.02.033 (2018).

[5] Cerquitelli, T. Predicting large scale fine grain energy consumption. *J. Energy Procedia*. **111**, 1079–1088. 10.1016/j.egypro.2017.03.271 (2017).

[6] Tang, Z. et al. Rice threshing state prediction of threshing cylinder undergoing unbalanced harmonic response. *Comput. Electron. Agric.* **204**, 107547. 10.1016/j.compag.2022.107547 (2023).

[7] Liang, Z., Li, Y., De Baerdemaeker, J., Xu, L. & Saeys, W. Development and testing of a multi-duct cleaning device for tangential-longitudinal flow rice combine harvesters. *Biosyst. Eng.* **182**, 95–106. 10.1016/j.biosystemseng.2019.04.004 (2019).

[8] Imthiyas, A., Saravanan, M., Kumar, P., Richard Meclar, F. & Satyanarayan, D. K. Design of muskmelon seed peeling machine. *IOP Conf. Series: Mater. Sci. Eng.* **993**, 012032. 10.1088/1757-899X/993/1/012032 (2020).

[9] Du, Z., Hu, Y. & Buttar, N. A. Analysis of mechanical properties for tea stem using grey relational analysis coupled with multiple linear regression. *Sci. Hort.* **260**, 108886. 10.1016/j.scienta.2019.108886 (2020).

[10] Xue, K. et al. Biomechanical modeling of rice seedling stalk based on multi-scale structure and heterogeneous materials. *Comput. Electron. Agric.* **210**, 107904. 10.1016/j.compag.2023.107904 (2023).

[11] Yang, R., Chen, D., Zha, X., Pan, Z. & Shang, S. Optimization design and experiment of ear-picking and threshing devices of corn plot kernel harvester. *Agriculture* **11** (2021).

[12] Baruah, D. & Panesar, B. Energy requirement model for a combine harvester, part I: Development of component models. *J. Biosystems Eng.* **90**, 9–25. 10.1016/j.biosystemseng.2004.08.017 (2005).

[13] Zhou, X. et al. INMATEH Agricultural Eng., 10.35633/inmateh-67-49 (2022).

[14] Tang, Z., Zhang, B., Wang, M. & Zhang, H. Damping behaviour of a prestressed composite beam designed for the thresher of a combine harvester. *Biosyst. Eng.* **204**, 130–146. 10.1016/j.biosystemseng.2021.01.020 (2021).

[15] Simonyan, K. Development of a motorized stationary sorghum thresher. *Ama Agric. Mech. Asia Afr. Latin Am.* **40**, 47 (2009).

[16] Jiangtao, J., Jingpeng, H., Shengsheng, W., Ruihong, Z. & Jing, P. Vibration and impact detection of axial-flow threshing unit under dynamic threshing conditions. *INMATEH-Agricultural Eng.* **60**10.35633/inmateh-60-21 (2020).

[17] Bhardwaj, M., Dogra, R., Javed, M., Singh, M. & Dogra, B. Optimization of conventional combine harvester to reduce combine losses for basmati rice (Oryza Sativa). *Agric. Sci.* **12**, 259–272. 10.4236/as.2021.123017 (2021).

[18] Chethan, C. R., Baldev Dogra, R. D. & Singh, D. Detection of crop flow path in axial flow paddy thresher by using magnetometer. *Futuristic Trends Robot. Autom.* **3**, 126–136 (2024).

[19] Tang, X. H. et al. Research status of threshing and separation device of combine harvester in China. *Res. Agric. Mech.* **44**, 1–9 (2022).

[20] Yang, L. et al. Improved design and bench test based on tangential flow-transverse axial flow maize threshing system. *Trans. Chin. Soc. Agric. Eng.* **34**, 35–43 (2018).

[21] Tang, Z., He, J. Z., Zhou, Y. P. & Liu, J. B. Threshing parameter test and analysis of multi drum threshing and separation device. *Res. Agric. Mech.* **37**, 153–157 (2017).

[22] Miu, P. I. & Kutzbach, H. D. Mathematical model of material kinematics in an axial threshing unit. *J. Comput. Electron. Agric.* **58**, 93–99. 10.1016/j.compag.2007.04.002 (2007).

[23] Addo, A., Bart-Plange, A., Asuboah, R. & Dzisi, K. Effect of different threshing cylinders on soybean quality. *J. Sci. Technol.* **24**, 121–125. 10.4314/just.v24i2.32924 (2004).

[24] Khazaei, J., Mohtasebi, S. & Rajabi Pour, A. Behroozi Lar, M. Determining the force and energy required for picking Chickpea pod as a criterion for Estimation of resistance to shatter. *Iran. J. Agricultural Sci.* **35**, 517–529 (2004).

[25] Liu, Y. et al. Development of a variable-diameter threshing drum for rice combine harvester using MBD - DEM coupling simulation. *Comput. Electron. Agric.* **196**, 106859. 10.1016/j.compag.2022.106859 (2022).

[26] Su, Z., Ding, Z., Tian, L., Lin, X. & Wang, Z. Design and performance test of variable diameter threshing drum of combine harvester. *Food Sci. Nutr.* **9**, 4322–4334. 10.1002/fsn3.2402 (2021). 34401082 10.1002/fsn3.2402 PMC8358346

[27] Zhalnin, E. & Chaplygin, M. Dynamics of fractional composition of Grain-and-Straw mass being threshedin the threshing mechanism of a combine harvester. *Eng. Technol. Syst.* **32**, 249–262. 10.15507/2658-4123.032.202202.249-262 (2022).

[28] Asli-Ardeh, E. & Yousef, A. Study of performance parameters of threshing unit in a single plant thresher. *Am. J. Agric. Biol. Sci.* **4**, 92–96. 10.3844/ajabssp.2009.92.96 (2009).

[29] Gummert, M., Kutzbach, H., Mühlbauer, W., Wacker, P. & Quick, G. Performance evaluation of an IRRI axial-flow paddy thresher. *Agric. Mech. Asia Afr. Latin Am.* **23**, 47–54 (1992).

[30] Singh, K., Poddar, R. R., Agrawal, K., Hota, S. & Singh, M. K. Development and evaluation of multi millet thresher. *J. Appl. Nat. Sci.* **7**, 939–948. 10.31018/jans.v7i2.711 (2015).

[31] Helmy, M., Yousef, I. & Badawy, M. Performance evaluation of some sunflower threshers. *Egypt. J. Agric. Res.* **78**, 959–975. 10.21608/ejar.2000.322529 (2000).

[32] Asli-Ardeh, E. A. & Abbaspour-Gilandeh, Y. Investigation of the effective factors on threshing loss, damaged grains percent and material other than grain to grain ratio on an auto head feed threshing unit. *Am. J. Agric. Biol. Sci.* **3**, 699–705. 10.3844/ajabssp.2008.699.705 (2008).

[33] El-Haddad, W. A simplified design and performance study of threshing and winnowing machine suitable for sample holdings (Tanta Univ., 2000).

[34] Abdeen, M. A., Salem, A. E. & Zhang, G. Longitudinal axial flow rice thresher performance optimization using the taguchi technique. *Agriculture* **11** (2021).

[35] Palanikumar, K. Experimental investigation and optimisation in drilling of GFRP composites. *J. Meas.* **44**, 2138–2148. 10.1016/j.measurement.2011.07.023 (2011).

[36] Asiltürk, I. & Akkuş, H. Determining the effect of cutting parameters on surface roughness in hard turning using the Taguchi method. *J. Meas.* **44**, 1697–1704. 10.1016/j.measurement.2011.07.003 (2011).

[37] Abdeen, M. A., Xie, G., Salem, A. E., Fu, J. & Zhang, G. Longitudinal axial flow rice thresher feeding rate monitoring based on force sensing resistors. *Sci. Rep.* **12**, 1369. 10.1038/s41598-021-04675-w (2022). 35079018 10.1038/s41598-021-04675-w PMC8789848

[38] Gbabo, A., Gana, I. M. & Amoto, M. S. Design, fabrication and testing of a millet thresher. *Net J. Agric. Sci.* **1**, 100–106 (2013). http://repository.futminna.edu.ng:8080/jspui/handle/123456789/13404

[39] Osueke, E. Study of the influence of crop, machine and operating parameters on performance of cereal threshers. *Int. J. Eng. Res. Dev.* **7**, 1–9 (2014).

[40] Ahuja, M., Dogra, B., Narang, M. & Dogra, R. Development and evaluation of axial flow paddy thresher equipped with feeder chain type mechanical feeding system. *Curr. J. Appl. Sci. Technol.* **23**, 1–10. 10.9734/CJAST/2017/35696 (2017).

[41] Abdollahpour, S. & Ghassemzadeh, H. Designing, fabrication and evaluation of threshing unit edible sunflower. *Agricultural Eng. International: CIGR J.* **21**, 52–58 (2019).

[42] Sudajan, S., Salokhe, V. & Triratanasirichai, K. Power and machinery: Effect of type of drum, drum speed and feed rate on sunflower threshing. *Biosyst. Eng.* **83**, 413–421. 10.1006/bioe.2002.0133 (2002).

[43] Li, X., Zhang, W., Wang, W. & Huang, Y. Design and test of longitudinal axial flow staggered millet flexible threshing device. *Agriculture* **12**, 1179 (2022). https://www.mdpi.com/2077-0472/12/8/1179#

[44] Esgici, R., Pekitkan, F. G. & Sessiz, A. Evaluation of cylinder rotational speed for rice grain losses and broken grain ratio. *J. Agric. Mach. Sci.* **16**, 28–33 (2020).

[45] Hailemesikel, S. T. et al. Effects of machine-crop parameters on mechanical grain damage in rice threshing. *Cogent Food Agric.* **10**, 2367381. 10.1080/23311932.2024.2367381 (2024).

[46] Ahmed, R. Evaluation of local machine performance for faba bean curshing. *Misr J. Agric. Eng.* **34**, 1167–1178. 10.21608/mjae.2017.97449 (2017).

[47] El Ghobashy, H. et al. Development and evaluation of a dual-purpose machine for chopping and crushing forage crops. *Heliyon* **9**10.1016/j.heliyon.2023.e15460 (2023). 10.1016/j.heliyon.2023.e15460 PMC10131070 37123933

